# 5,22-Stigmastadien-3β-yl *p*-toluene­sulfonate

**DOI:** 10.1107/S1600536810016661

**Published:** 2010-05-15

**Authors:** Kamal Aziz Ketuly, A. Hamid A. Hadi, Hamid Khaledi, Edward R. T. Tiekink

**Affiliations:** a Department of Chemistry, University of Malaya, 50603 Kuala Lumpur, Malaysia

## Abstract

The asymmetric unit of the title compound {systematic name: (3*S*,8*S*,9*S*,10*R*,13*R*,14*S*,17*R*)-17-[(*E*,2*R*,5*S*)-5-ethyl-6-methyl­hept-3-en-2-yl]-10,13-dimethyl-2,3,4,7,8,9,11,12,14,15,16,17-dodeca­hydro-1*H*-cyclo­penta­[*a*]phenanthren-3-yl *p*-toluene­sulfonate}, C_36_H_54_O_3_S, comprises two independent mol­ecules that differ significantly in terms of the relative orientations of the peripheral groups; the conformation about the C=C bond of the side chain is *E*. In the crystal, mol­ecules associate into linear supra­molecular chains aligned along the *a* axis *via* C—H⋯O inter­actions.

## Related literature

For the use of 5,22-stigmastadien-3β-yl *p*-toluene­sulfonate, see: Partridge *et al.* (1974 ▶); Khripach *et al.* (2002 ▶); Foley *et al.* (2010 ▶); Ketuly *et al.* (1997 ▶). For the synthesis, see: Foley *et al.* (2010 ▶).
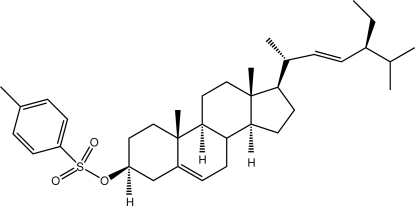


## Experimental

### 

#### Crystal data

C_36_H_54_O_3_S
*M* *_r_* = 566.86
Triclinic, 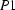

*a* = 7.0361 (1) Å
*b* = 11.2350 (1) Å
*c* = 21.1550 (2) Å
α = 90.777 (1)°
β = 96.166 (1)°
γ = 101.153 (1)°
*V* = 1630.23 (3) Å^3^
*Z* = 2
Mo *K*α radiation
μ = 0.13 mm^−1^
*T* = 100 K
0.49 × 0.37 × 0.27 mm

#### Data collection

Bruker SMART APEX CCD diffractometer
Absorption correction: multi-scan (*SADABS*; Sheldrick, 1996 ▶) *T*_min_ = 0.669, *T*_max_ = 0.746
15647 measured reflections
13115 independent reflections
12382 reflections with *I* > 2σ(*I*)
*R*_int_ = 0.018

#### Refinement

*R*[*F* ^2^ > 2σ(*F* ^2^)] = 0.035
*wR*(*F* ^2^) = 0.089
*S* = 1.02
13115 reflections
735 parameters
3 restraints
H-atom parameters constrained
Δρ_max_ = 0.35 e Å^−3^
Δρ_min_ = −0.37 e Å^−3^
Absolute structure: Flack (1983 ▶), 5643 Friedel pairs
Flack parameter: 0.02 (4)

### 

Data collection: *APEX2* (Bruker, 2008 ▶); cell refinement: *SAINT* (Bruker, 2008 ▶); data reduction: *SAINT*; program(s) used to solve structure: *SHELXS97* (Sheldrick, 2008 ▶); program(s) used to refine structure: *SHELXL97* (Sheldrick, 2008 ▶); molecular graphics: *ORTEP-3* (Farrugia, 1997 ▶), *DIAMOND* (Brandenburg, 2006 ▶) and *Qmol* (Gans & Shalloway, 2001 ▶); software used to prepare material for publication: *publCIF* (Westrip, 2010 ▶).

## Supplementary Material

Crystal structure: contains datablocks global, I. DOI: 10.1107/S1600536810016661/hb5438sup1.cif

Structure factors: contains datablocks I. DOI: 10.1107/S1600536810016661/hb5438Isup2.hkl

Additional supplementary materials:  crystallographic information; 3D view; checkCIF report

## Figures and Tables

**Table 1:** Hydrogen-bond geometry (Å, °)

*D*—H⋯*A*	*D*—H	H⋯*A*	*D*⋯*A*	*D*—H⋯*A*
C7—H7⋯O3^i^	0.95	2.49	3.217 (2)	134
C13—H13*B*⋯O6	0.99	2.54	3.519 (2)	172
C40—H40⋯O5^ii^	0.95	2.48	3.193 (2)	131
C42—H42*A*⋯O2	0.99	2.56	3.548 (2)	175
C44*A*—H44*C*⋯O2	0.98	2.54	3.390 (2)	145

Symmetry codes: (i) 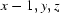; (ii) 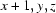.

**Fig. 1.**
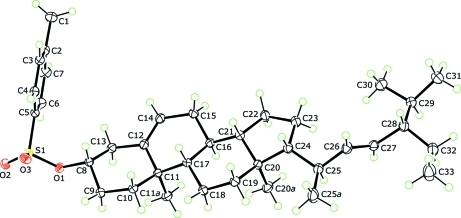
The molecular structure of the first independent molecule in (I) displacement ellipsoids at the 50% probability level.

**Fig. 2.**
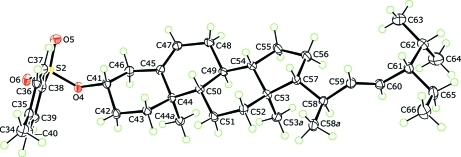
The molecular structure of the second independent molecule in (I) showing displacement ellipsoids at the 50% probability level.

**Fig. 3.**
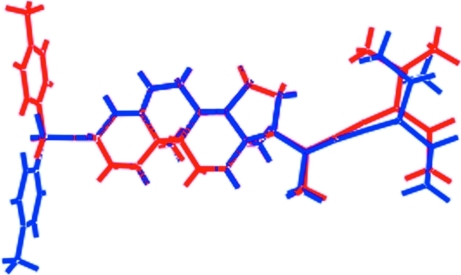
Overlap diagram highlighting the different conformations of the first (red) and second (blue) independent molecules in (I).

**Fig. 4.**
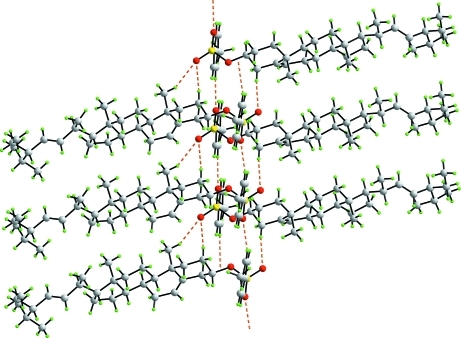
A view of a supramolecular chain in (I) aligned along the a axis. The C–H···O contacts are shown as orange dashed lines. Colour code: O, red; C, grey; and H, green.

**Fig. 5.**
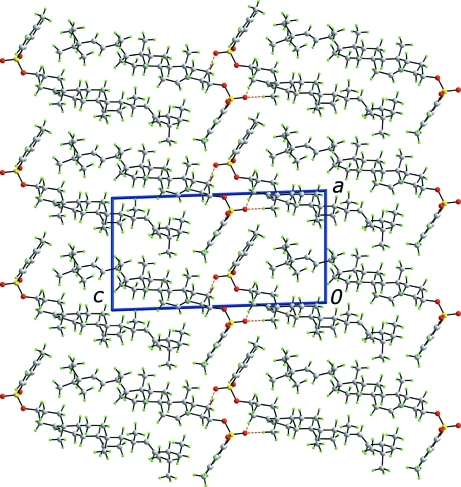
A view in projection down the a axis of the unit cell contents in (I) showing the inter-digitation of supramolecular chains. The C–H···O contacts are shown as orange dashed lines, respectively. Colour code: O, red; C, grey; and H, green.

## supplementary crystallographic information

### Comment

The title compound, (I), a stigmasterol tosylate, has been used as a precursor for the synthesis of cholesterol and other sterols with variable side-chains (Partridge *et al.* 1974; Khripach *et al.* 2002; Foley *et al.* 2010) and for the determination of absolute configuration (Ketuly *et al.* 1997). Herein, (I) has been characterised by crystallography and shown to crystallise with two independent molecules in the crystallographic asymmetric unit, Figs 1 and 2. While the central steroid residues in the independent molecules are virtually superimposable, as seen from Fig. 3, the peripheral groups have quite different orientations. These differences are quantified in the values of the C5–S1–O1–C8 and C38–S2–O4–C41 torsion angles of 67.92 (13) and -63.53 (14) °, respectively, which indicate that the tosylate groups lie to either side of the respective molecule. The differences in the orientation of the terminal *iso*-propyl group are best quantified in the C27–C28–C29–C30, C31 torsion angles of 45.2 (2) and 168.45 (16) °, respectively, compared with 58.2 (2) and -66.8 (2) ° for C60–C61–C62–C63,C64, respectively. The conformation about the C═C [C26═C27 = 1.322 (3) Å and C59═C60 = 1.324 (2) Å] double bond of the side-chain in each molecule is *E*.

In the crystal structure, the molecules associate into linear supramolecular chains aligned along the *a* axis mediated by C–H···O interactions, Fig. 2 and Table 1. The chains inter-digitate along the *b* axis as shown in Fig. 5.

### Experimental

Stigmasterol (40 g, 97 mmol) was dissolved in dried and redistilled pyridine (250 ml) and *p*-toluenesulfonyl chloride (30 g, 157.4 mmol) added, and the solution was then stirred at room temperature for 28 h. The reaction mixture was mixed with 5% aqueous sodium bicarbonate and the solid precipitate filtered, washed with water, dried at room temperature, and recovered (52 g). The crude product was recrystallized from acetone, yielding fine crystals of stigmasterol tosylate (45.8 g), m.pt. 407–410 K. The compound was further recrystallised and colourless blocks of (I) were grown from n-hexane:carbon tetrachloride (1/1), m.pt. 415–417 K [Lit. 415–417 K (Foley *et al.* , 2010)].

### Refinement

The H atoms were geometrically placed (C–H = 0.95–1.00 Å) and refined as riding with *U_iso_*(H) = 1.2–1.5*U_eq_*(C).

### Crystal data

**Table:**

C_36_H_54_O_3_S	*Z* = 2
*M**_r_* = 566.86	*F*(000) = 620
Triclinic, *P*1	*D*_x_ = 1.155 Mg m^−^^3^
Hall symbol: P 1	Mo *K*α radiation, λ = 0.71073 Å
*a* = 7.0361 (1) Å	Cell parameters from 8182 reflections
*b* = 11.2350 (1) Å	θ = 2.6–30.5°
*c* = 21.1550 (2) Å	µ = 0.13 mm^−^^1^
α = 90.777 (1)°	*T* = 100 K
β = 96.166 (1)°	Block, colourless
γ = 101.153 (1)°	0.49 × 0.37 × 0.27 mm
*V* = 1630.23 (3) Å^3^	

### Data collection

**Table:**

Bruker SMART APEX CCD diffractometer	13115 independent reflections
Radiation source: fine-focus sealed tube	12382 reflections with *I* > 2σ(*I*)
graphite	*R*_int_ = 0.018
ω scans	θ_max_ = 27.5°, θ_min_ = 1.9°
Absorption correction: multi-scan (*SADABS*; Sheldrick, 1996)	*h* = −9→9
*T*_min_ = 0.669, *T*_max_ = 0.746	*k* = −14→14
15647 measured reflections	*l* = −27→27

### Refinement

**Table:**

Refinement on *F*^2^	Secondary atom site location: difference Fourier map
Least-squares matrix: full	Hydrogen site location: inferred from neighbouring sites
*R*[*F*^2^ > 2σ(*F*^2^)] = 0.035	H-atom parameters constrained
*wR*(*F*^2^) = 0.089	*w* = 1/[σ^2^(*F*_o_^2^) + (0.049*P*)^2^ + 0.2642*P*] where *P* = (*F*_o_^2^ + 2*F*_c_^2^)/3
*S* = 1.02	(Δ/σ)_max_ = 0.001
13115 reflections	Δρ_max_ = 0.35 e Å^−^^3^
735 parameters	Δρ_min_ = −0.37 e Å^−^^3^
3 restraints	Absolute structure: Flack (1983), 5643 Friedel pairs
Primary atom site location: structure-invariant direct methods	Flack parameter: 0.02 (4)

### Special details

**Table:**

Geometry. All s.u.'s (except the s.u. in the dihedral angle between two l.s. planes) are estimated using the full covariance matrix. The cell s.u.'s are taken into account individually in the estimation of s.u.'s in distances, angles and torsion angles; correlations between s.u.'s in cell parameters are only used when they are defined by crystal symmetry. An approximate (isotropic) treatment of cell s.u.'s is used for estimating s.u.'s involving l.s. planes.
Refinement. Refinement of *F*^2^ against ALL reflections. The weighted *R*-factor wR and goodness of fit *S* are based on *F*^2^, conventional *R*-factors *R* are based on *F*, with *F* set to zero for negative *F*^2^. The threshold expression of *F*^2^ > 2σ(*F*^2^) is used only for calculating *R*-factors(gt) etc. and is not relevant to the choice of reflections for refinement. *R*-factors based on *F*^2^ are statistically about twice as large as those based on *F*, and *R*- factors based on ALL data will be even larger.

### Fractional atomic coordinates and isotropic or equivalent isotropic displacement parameters (Å^2^)

**Table:**

	*x*	*y*	*z*	*U*_iso_*/*U*_eq_	
S1	0.69443 (5)	0.84972 (3)	0.44298 (2)	0.01550 (9)	
O1	0.67495 (19)	0.97242 (11)	0.47537 (6)	0.0185 (3)	
O2	0.62261 (19)	0.85861 (12)	0.37793 (6)	0.0202 (3)	
O3	0.88690 (18)	0.82684 (12)	0.45847 (6)	0.0213 (3)	
C1	0.1261 (3)	0.48353 (19)	0.57090 (10)	0.0283 (5)	
H1A	0.1484	0.5009	0.6169	0.042*	
H1B	0.1450	0.4011	0.5619	0.042*	
H1C	−0.0074	0.4903	0.5551	0.042*	
C2	0.2679 (3)	0.57305 (17)	0.53824 (9)	0.0208 (4)	
C3	0.4643 (3)	0.56629 (17)	0.54289 (9)	0.0190 (4)	
H3	0.5088	0.5034	0.5663	0.023*	
C4	0.5962 (3)	0.65025 (16)	0.51380 (8)	0.0174 (4)	
H4	0.7301	0.6452	0.5172	0.021*	
C5	0.5300 (3)	0.74157 (16)	0.47974 (8)	0.0155 (3)	
C6	0.3344 (3)	0.74989 (18)	0.47399 (10)	0.0214 (4)	
H6	0.2903	0.8125	0.4503	0.026*	
C7	0.2052 (3)	0.66572 (18)	0.50327 (10)	0.0240 (4)	
H7	0.0713	0.6709	0.4996	0.029*	
C8	0.7474 (3)	0.99334 (17)	0.54366 (8)	0.0172 (4)	
H8	0.7888	0.9189	0.5611	0.021*	
C9	0.9196 (3)	1.09800 (17)	0.54931 (9)	0.0206 (4)	
H9A	0.8814	1.1693	0.5282	0.025*	
H9B	1.0263	1.0754	0.5278	0.025*	
C10	0.9908 (3)	1.13062 (17)	0.61942 (9)	0.0193 (4)	
H10A	1.0969	1.2032	0.6220	0.023*	
H10B	1.0467	1.0630	0.6382	0.023*	
C11	0.8321 (3)	1.15659 (15)	0.66011 (8)	0.0171 (4)	
C11A	0.7752 (3)	1.27858 (16)	0.64174 (9)	0.0223 (4)	
H11A	0.7340	1.2768	0.5959	0.034*	
H11B	0.8878	1.3448	0.6524	0.034*	
H11C	0.6678	1.2917	0.6652	0.034*	
C12	0.6484 (3)	1.05841 (16)	0.64686 (9)	0.0172 (4)	
C13	0.5805 (3)	1.02109 (17)	0.57757 (9)	0.0198 (4)	
H13A	0.4748	0.9483	0.5752	0.024*	
H13B	0.5277	1.0874	0.5561	0.024*	
C14	0.5425 (3)	1.01280 (16)	0.69279 (8)	0.0192 (3)	
H14	0.4307	0.9513	0.6808	0.023*	
C15	0.5868 (3)	1.05133 (16)	0.76192 (8)	0.0190 (3)	
H15A	0.6230	0.9829	0.7863	0.023*	
H15B	0.4675	1.0697	0.7775	0.023*	
C16	0.7520 (3)	1.16293 (15)	0.77506 (8)	0.0162 (3)	
H16	0.6978	1.2374	0.7652	0.019*	
C17	0.9115 (3)	1.15600 (16)	0.73162 (8)	0.0161 (3)	
H17	0.9478	1.0749	0.7383	0.019*	
C18	1.0995 (3)	1.25072 (17)	0.74971 (9)	0.0205 (4)	
H18A	1.0768	1.3311	0.7359	0.025*	
H18B	1.2035	1.2301	0.7261	0.025*	
C19	1.1718 (3)	1.26021 (17)	0.82118 (9)	0.0204 (4)	
H19A	1.2166	1.1848	0.8339	0.024*	
H19B	1.2843	1.3287	0.8295	0.024*	
C20	1.0109 (3)	1.27989 (15)	0.86124 (8)	0.0162 (3)	
C20A	0.9540 (3)	1.40312 (16)	0.84761 (9)	0.0226 (4)	
H20A	1.0693	1.4680	0.8565	0.034*	
H20B	0.8548	1.4158	0.8748	0.034*	
H20C	0.9016	1.4041	0.8028	0.034*	
C21	0.8384 (3)	1.17209 (16)	0.84454 (8)	0.0167 (3)	
H21	0.8924	1.0969	0.8523	0.020*	
C22	0.7035 (3)	1.17900 (18)	0.89584 (9)	0.0234 (4)	
H22A	0.6270	1.0977	0.9032	0.028*	
H22B	0.6127	1.2342	0.8838	0.028*	
C23	0.8437 (3)	1.22925 (17)	0.95564 (9)	0.0217 (4)	
H23A	0.8421	1.1665	0.9881	0.026*	
H23B	0.8038	1.3007	0.9742	0.026*	
C24	1.0514 (3)	1.26601 (17)	0.93432 (9)	0.0172 (4)	
H24	1.1180	1.1955	0.9412	0.021*	
C25	1.1738 (3)	1.37487 (16)	0.97497 (8)	0.0210 (4)	
H25	1.1100	1.4463	0.9670	0.025*	
C25A	1.3849 (3)	1.4103 (2)	0.95873 (10)	0.0347 (5)	
H25A	1.4560	1.4774	0.9873	0.052*	
H25B	1.3844	1.4357	0.9146	0.052*	
H25C	1.4488	1.3403	0.9640	0.052*	
C26	1.1761 (3)	1.34890 (17)	1.04477 (9)	0.0207 (4)	
H26	1.2346	1.2838	1.0599	0.025*	
C27	1.1020 (3)	1.41072 (16)	1.08631 (9)	0.0188 (4)	
H27	1.0390	1.4731	1.0699	0.023*	
C28	1.1069 (3)	1.39278 (16)	1.15671 (8)	0.0198 (4)	
H28	1.1668	1.3206	1.1670	0.024*	
C29	0.9004 (3)	1.36906 (17)	1.17799 (8)	0.0216 (4)	
H29	0.8534	1.4476	1.1767	0.026*	
C30	0.7557 (3)	1.2771 (2)	1.13373 (10)	0.0328 (5)	
H30A	0.8060	1.2021	1.1302	0.049*	
H30B	0.7384	1.3109	1.0915	0.049*	
H30C	0.6300	1.2594	1.1511	0.049*	
C31	0.8994 (3)	1.3254 (2)	1.24636 (10)	0.0323 (5)	
H31A	0.7700	1.3225	1.2601	0.048*	
H31B	0.9967	1.3818	1.2746	0.048*	
H31C	0.9309	1.2442	1.2481	0.048*	
C32	1.2370 (3)	1.50536 (19)	1.19232 (9)	0.0277 (4)	
H32A	1.1836	1.5780	1.1799	0.033*	
H32B	1.2307	1.4964	1.2386	0.033*	
C33	1.4489 (3)	1.5262 (3)	1.17963 (12)	0.0451 (6)	
H33A	1.5061	1.4574	1.1946	0.068*	
H33B	1.5207	1.6009	1.2022	0.068*	
H33C	1.4568	1.5338	1.1339	0.068*	
S2	0.30849 (5)	1.27220 (3)	0.44943 (2)	0.01603 (9)	
O4	0.32421 (19)	1.14814 (11)	0.41769 (6)	0.0179 (3)	
O5	0.11769 (19)	1.29670 (12)	0.43282 (6)	0.0219 (3)	
O6	0.3765 (2)	1.26274 (12)	0.51495 (6)	0.0215 (3)	
C34	0.8898 (3)	1.64290 (19)	0.32767 (10)	0.0264 (4)	
H34A	1.0067	1.6706	0.3577	0.040*	
H34B	0.8316	1.7125	0.3147	0.040*	
H34C	0.9255	1.6041	0.2901	0.040*	
C35	0.7448 (3)	1.55291 (17)	0.35912 (9)	0.0203 (4)	
C36	0.5459 (3)	1.55371 (17)	0.34939 (9)	0.0198 (4)	
H36	0.5015	1.6139	0.3238	0.024*	
C37	0.4116 (3)	1.46780 (17)	0.37652 (9)	0.0182 (4)	
H37	0.2764	1.4690	0.3695	0.022*	
C38	0.4769 (3)	1.38034 (16)	0.41391 (9)	0.0164 (4)	
C39	0.6743 (3)	1.37796 (17)	0.42510 (9)	0.0192 (4)	
H39	0.7182	1.3183	0.4512	0.023*	
C40	0.8067 (3)	1.46471 (18)	0.39740 (9)	0.0213 (4)	
H40	0.9419	1.4637	0.4048	0.026*	
C41	0.2679 (3)	1.12867 (16)	0.34818 (8)	0.0170 (4)	
H41	0.2172	1.2001	0.3306	0.020*	
C42	0.4461 (3)	1.11455 (17)	0.31752 (9)	0.0179 (4)	
H42A	0.5007	1.0467	0.3366	0.021*	
H42B	0.5467	1.1897	0.3250	0.021*	
C43	0.3909 (3)	1.08909 (16)	0.24600 (9)	0.0179 (4)	
H43A	0.5085	1.0780	0.2266	0.022*	
H43B	0.3480	1.1610	0.2271	0.022*	
C44	0.2286 (2)	0.97669 (16)	0.22859 (8)	0.0154 (3)	
C44A	0.3072 (3)	0.86021 (16)	0.24517 (9)	0.0183 (4)	
H44A	0.4214	0.8581	0.2227	0.027*	
H44B	0.2057	0.7888	0.2321	0.027*	
H44C	0.3443	0.8600	0.2912	0.027*	
C45	0.0589 (3)	0.98035 (16)	0.26753 (8)	0.0160 (3)	
C46	0.1093 (3)	1.01536 (17)	0.33770 (9)	0.0193 (4)	
H46A	0.1538	0.9475	0.3604	0.023*	
H46B	−0.0088	1.0300	0.3556	0.023*	
C47	−0.1263 (2)	0.94840 (16)	0.24258 (8)	0.0195 (4)	
H47	−0.2239	0.9535	0.2697	0.023*	
C48	−0.1933 (3)	0.90488 (18)	0.17491 (9)	0.0224 (4)	
H48A	−0.2561	0.9667	0.1527	0.027*	
H48B	−0.2928	0.8293	0.1748	0.027*	
C49	−0.0279 (2)	0.88027 (17)	0.13799 (8)	0.0169 (4)	
H49	0.0021	0.7995	0.1499	0.020*	
C50	0.1550 (2)	0.97888 (16)	0.15671 (8)	0.0152 (3)	
H50	0.1138	1.0586	0.1502	0.018*	
C51	0.3161 (3)	0.97594 (17)	0.11341 (8)	0.0195 (4)	
H51A	0.3752	0.9043	0.1234	0.023*	
H51B	0.4192	1.0493	0.1231	0.023*	
C52	0.2445 (3)	0.97042 (16)	0.04174 (8)	0.0192 (4)	
H52A	0.2008	1.0467	0.0301	0.023*	
H52B	0.3534	0.9627	0.0170	0.023*	
C53	0.0754 (2)	0.86221 (15)	0.02491 (8)	0.0154 (3)	
C53A	0.1438 (3)	0.74110 (16)	0.03621 (9)	0.0198 (4)	
H53A	0.1851	0.7351	0.0816	0.030*	
H53B	0.2536	0.7378	0.0118	0.030*	
H53C	0.0363	0.6734	0.0224	0.030*	
C54	−0.0868 (2)	0.87808 (16)	0.06625 (8)	0.0175 (4)	
H54	−0.1178	0.9596	0.0566	0.021*	
C55	−0.2647 (3)	0.78459 (19)	0.03754 (9)	0.0265 (4)	
H55A	−0.3873	0.8112	0.0444	0.032*	
H55B	−0.2656	0.7044	0.0565	0.032*	
C56	−0.2399 (3)	0.77885 (19)	−0.03430 (9)	0.0254 (4)	
H56A	−0.3447	0.8107	−0.0595	0.030*	
H56B	−0.2456	0.6940	−0.0489	0.030*	
C57	−0.0379 (3)	0.85800 (17)	−0.04245 (9)	0.0179 (4)	
H57	−0.0587	0.9421	−0.0512	0.021*	
C58	0.0537 (3)	0.81453 (16)	−0.09941 (8)	0.0186 (3)	
H58	0.0752	0.7303	−0.0915	0.022*	
C58A	0.2503 (3)	0.89545 (18)	−0.10828 (9)	0.0248 (4)	
H58A	0.2919	0.8736	−0.1489	0.037*	
H58B	0.3478	0.8838	−0.0734	0.037*	
H58C	0.2370	0.9807	−0.1082	0.037*	
C59	−0.0795 (3)	0.81142 (16)	−0.16047 (8)	0.0195 (4)	
H59	−0.1195	0.8850	−0.1721	0.023*	
C60	−0.1459 (3)	0.71560 (17)	−0.19935 (9)	0.0189 (4)	
H60	−0.1182	0.6399	−0.1857	0.023*	
C61	−0.2616 (3)	0.71673 (16)	−0.26345 (9)	0.0207 (4)	
H61	−0.2784	0.8022	−0.2703	0.025*	
C62	−0.4672 (3)	0.63669 (18)	−0.26825 (10)	0.0252 (4)	
H62	−0.5354	0.6497	−0.3108	0.030*	
C63	−0.5848 (3)	0.6760 (2)	−0.21817 (11)	0.0367 (5)	
H63A	−0.7194	0.6306	−0.2254	0.055*	
H63B	−0.5839	0.7631	−0.2211	0.055*	
H63C	−0.5268	0.6595	−0.1758	0.055*	
C64	−0.4669 (3)	0.50155 (19)	−0.26452 (12)	0.0360 (5)	
H64A	−0.4080	0.4846	−0.2223	0.054*	
H64B	−0.3912	0.4774	−0.2971	0.054*	
H64C	−0.6011	0.4556	−0.2718	0.054*	
C65	−0.1447 (3)	0.68280 (19)	−0.31588 (9)	0.0284 (4)	
H65A	−0.0975	0.6076	−0.3044	0.034*	
H65B	−0.2323	0.6655	−0.3562	0.034*	
C66	0.0285 (3)	0.7812 (2)	−0.32658 (10)	0.0336 (5)	
H66A	−0.0170	0.8557	−0.3387	0.050*	
H66B	0.0962	0.7544	−0.3607	0.050*	
H66C	0.1183	0.7971	−0.2873	0.050*	

### Atomic displacement parameters (Å^2^)

**Table:**

	*U*^11^	*U*^22^	*U*^33^	*U*^12^	*U*^13^	*U*^23^
S1	0.0165 (2)	0.0165 (2)	0.0130 (2)	0.00202 (16)	0.00175 (15)	0.00057 (16)
O1	0.0251 (7)	0.0152 (6)	0.0144 (7)	0.0033 (5)	−0.0008 (5)	0.0006 (5)
O2	0.0240 (7)	0.0214 (7)	0.0139 (7)	0.0021 (5)	0.0004 (5)	0.0000 (5)
O3	0.0172 (7)	0.0258 (7)	0.0209 (7)	0.0030 (5)	0.0039 (5)	0.0028 (6)
C1	0.0279 (11)	0.0257 (10)	0.0296 (11)	−0.0027 (8)	0.0108 (9)	0.0005 (9)
C2	0.0235 (10)	0.0180 (9)	0.0194 (10)	−0.0013 (7)	0.0056 (8)	−0.0040 (7)
C3	0.0246 (10)	0.0154 (9)	0.0165 (9)	0.0023 (7)	0.0026 (7)	−0.0002 (7)
C4	0.0179 (9)	0.0180 (9)	0.0160 (9)	0.0037 (7)	0.0009 (7)	−0.0007 (7)
C5	0.0164 (9)	0.0157 (8)	0.0138 (9)	0.0008 (7)	0.0027 (7)	−0.0001 (7)
C6	0.0155 (9)	0.0222 (10)	0.0267 (10)	0.0047 (7)	0.0007 (7)	0.0030 (8)
C7	0.0142 (9)	0.0255 (10)	0.0320 (11)	0.0027 (7)	0.0033 (8)	0.0000 (8)
C8	0.0215 (9)	0.0165 (9)	0.0120 (9)	0.0006 (7)	−0.0001 (7)	0.0002 (7)
C9	0.0237 (10)	0.0206 (9)	0.0149 (9)	−0.0023 (7)	0.0029 (7)	−0.0007 (7)
C10	0.0208 (9)	0.0180 (9)	0.0166 (9)	−0.0026 (7)	0.0031 (7)	−0.0013 (7)
C11	0.0198 (9)	0.0130 (8)	0.0161 (9)	−0.0013 (7)	0.0001 (7)	0.0012 (6)
C11A	0.0281 (10)	0.0172 (9)	0.0202 (9)	0.0015 (7)	0.0013 (7)	0.0008 (7)
C12	0.0188 (9)	0.0156 (8)	0.0167 (9)	0.0033 (7)	0.0005 (7)	−0.0010 (7)
C13	0.0212 (9)	0.0178 (9)	0.0182 (9)	−0.0008 (7)	0.0008 (7)	0.0000 (7)
C14	0.0173 (8)	0.0190 (8)	0.0194 (9)	−0.0007 (6)	0.0008 (7)	−0.0024 (7)
C15	0.0176 (9)	0.0210 (8)	0.0174 (9)	0.0004 (7)	0.0042 (7)	−0.0006 (7)
C16	0.0151 (8)	0.0152 (8)	0.0171 (9)	0.0007 (6)	0.0011 (7)	−0.0007 (6)
C17	0.0174 (9)	0.0153 (8)	0.0149 (8)	0.0005 (6)	0.0029 (7)	0.0006 (6)
C18	0.0199 (9)	0.0216 (9)	0.0169 (9)	−0.0035 (7)	0.0025 (7)	−0.0006 (7)
C19	0.0188 (9)	0.0226 (9)	0.0176 (9)	−0.0004 (7)	0.0008 (7)	−0.0006 (7)
C20	0.0179 (9)	0.0147 (8)	0.0149 (9)	0.0015 (7)	0.0004 (7)	−0.0002 (6)
C20A	0.0284 (10)	0.0172 (8)	0.0210 (9)	0.0051 (7)	−0.0033 (7)	−0.0003 (7)
C21	0.0181 (9)	0.0171 (8)	0.0149 (8)	0.0028 (7)	0.0031 (7)	0.0004 (6)
C22	0.0212 (9)	0.0288 (10)	0.0183 (9)	−0.0005 (7)	0.0046 (7)	−0.0043 (7)
C23	0.0228 (9)	0.0242 (9)	0.0172 (9)	0.0019 (7)	0.0040 (7)	−0.0014 (7)
C24	0.0184 (9)	0.0174 (9)	0.0159 (9)	0.0041 (7)	0.0009 (7)	0.0001 (7)
C25	0.0217 (9)	0.0216 (9)	0.0184 (9)	0.0015 (7)	0.0015 (7)	−0.0034 (7)
C25A	0.0263 (11)	0.0478 (13)	0.0241 (11)	−0.0071 (9)	0.0037 (8)	−0.0120 (9)
C26	0.0203 (9)	0.0213 (9)	0.0193 (9)	0.0044 (7)	−0.0033 (7)	−0.0012 (7)
C27	0.0191 (9)	0.0178 (9)	0.0178 (9)	0.0015 (7)	−0.0011 (7)	0.0007 (7)
C28	0.0227 (9)	0.0193 (8)	0.0178 (9)	0.0055 (7)	0.0019 (7)	−0.0007 (7)
C29	0.0235 (9)	0.0222 (9)	0.0178 (9)	0.0015 (7)	0.0021 (7)	0.0002 (7)
C30	0.0290 (11)	0.0367 (11)	0.0269 (11)	−0.0078 (9)	0.0038 (8)	−0.0029 (9)
C31	0.0363 (12)	0.0353 (11)	0.0225 (10)	−0.0018 (9)	0.0065 (9)	0.0018 (8)
C32	0.0286 (11)	0.0327 (11)	0.0194 (10)	0.0001 (8)	0.0027 (8)	−0.0040 (8)
C33	0.0288 (12)	0.0666 (17)	0.0329 (13)	−0.0074 (11)	0.0047 (10)	−0.0145 (11)
S2	0.0179 (2)	0.0153 (2)	0.0151 (2)	0.00321 (16)	0.00275 (16)	−0.00007 (16)
O4	0.0229 (7)	0.0170 (6)	0.0137 (6)	0.0044 (5)	0.0004 (5)	−0.0008 (5)
O5	0.0204 (7)	0.0202 (7)	0.0262 (8)	0.0048 (5)	0.0056 (6)	0.0024 (6)
O6	0.0286 (7)	0.0208 (7)	0.0151 (7)	0.0042 (6)	0.0031 (5)	−0.0006 (5)
C34	0.0282 (11)	0.0248 (10)	0.0256 (11)	0.0006 (8)	0.0090 (8)	0.0007 (8)
C35	0.0239 (10)	0.0187 (9)	0.0175 (9)	0.0008 (7)	0.0046 (7)	−0.0046 (7)
C36	0.0256 (10)	0.0189 (9)	0.0144 (9)	0.0045 (7)	0.0001 (7)	0.0000 (7)
C37	0.0185 (9)	0.0198 (9)	0.0160 (9)	0.0051 (7)	−0.0003 (7)	−0.0033 (7)
C38	0.0201 (9)	0.0137 (8)	0.0145 (9)	0.0010 (7)	0.0033 (7)	−0.0018 (7)
C39	0.0206 (9)	0.0179 (9)	0.0198 (9)	0.0065 (7)	0.0008 (7)	−0.0001 (7)
C40	0.0175 (9)	0.0216 (9)	0.0246 (10)	0.0038 (7)	0.0031 (7)	−0.0032 (7)
C41	0.0195 (9)	0.0169 (9)	0.0135 (9)	0.0028 (7)	−0.0018 (7)	−0.0004 (7)
C42	0.0167 (9)	0.0183 (9)	0.0170 (9)	−0.0006 (7)	0.0019 (7)	−0.0013 (7)
C43	0.0145 (9)	0.0194 (9)	0.0185 (9)	−0.0001 (7)	0.0019 (7)	−0.0015 (7)
C44	0.0156 (8)	0.0173 (8)	0.0131 (8)	0.0031 (7)	0.0006 (6)	−0.0005 (6)
C44A	0.0183 (9)	0.0186 (8)	0.0178 (9)	0.0050 (7)	−0.0016 (7)	−0.0005 (7)
C45	0.0175 (9)	0.0144 (8)	0.0158 (9)	0.0012 (6)	0.0040 (7)	−0.0003 (6)
C46	0.0191 (9)	0.0198 (9)	0.0185 (9)	0.0007 (7)	0.0051 (7)	0.0017 (7)
C47	0.0154 (8)	0.0242 (9)	0.0190 (9)	0.0022 (7)	0.0061 (7)	−0.0020 (7)
C48	0.0131 (8)	0.0319 (10)	0.0207 (9)	0.0010 (7)	0.0023 (7)	−0.0028 (7)
C49	0.0119 (8)	0.0222 (9)	0.0159 (9)	0.0024 (7)	0.0011 (6)	−0.0007 (7)
C50	0.0134 (8)	0.0159 (8)	0.0154 (9)	0.0015 (6)	−0.0001 (6)	0.0000 (6)
C51	0.0136 (9)	0.0252 (9)	0.0175 (9)	−0.0019 (7)	0.0019 (7)	−0.0019 (7)
C52	0.0200 (9)	0.0199 (9)	0.0158 (9)	−0.0012 (7)	0.0032 (7)	−0.0015 (7)
C53	0.0139 (8)	0.0166 (8)	0.0143 (8)	0.0010 (6)	−0.0007 (6)	0.0010 (6)
C53A	0.0207 (9)	0.0207 (9)	0.0187 (9)	0.0071 (7)	0.0006 (7)	0.0013 (7)
C54	0.0133 (8)	0.0206 (9)	0.0178 (9)	0.0034 (7)	−0.0012 (7)	−0.0009 (7)
C55	0.0152 (9)	0.0408 (11)	0.0201 (9)	−0.0015 (8)	−0.0006 (7)	−0.0052 (8)
C56	0.0163 (9)	0.0367 (11)	0.0209 (10)	0.0025 (8)	−0.0019 (7)	−0.0050 (8)
C57	0.0181 (9)	0.0197 (9)	0.0154 (9)	0.0054 (7)	−0.0024 (7)	−0.0019 (7)
C58	0.0214 (9)	0.0181 (8)	0.0167 (9)	0.0067 (7)	−0.0005 (7)	−0.0026 (6)
C58A	0.0224 (10)	0.0318 (10)	0.0189 (9)	0.0017 (8)	0.0033 (7)	−0.0055 (7)
C59	0.0237 (9)	0.0181 (8)	0.0173 (9)	0.0072 (7)	−0.0001 (7)	−0.0002 (7)
C60	0.0185 (9)	0.0175 (8)	0.0204 (9)	0.0045 (7)	−0.0002 (7)	0.0000 (7)
C61	0.0218 (9)	0.0193 (8)	0.0193 (9)	0.0029 (7)	−0.0030 (7)	−0.0028 (7)
C62	0.0197 (9)	0.0284 (10)	0.0262 (10)	0.0036 (8)	−0.0006 (7)	−0.0034 (8)
C63	0.0240 (11)	0.0482 (13)	0.0390 (13)	0.0088 (9)	0.0057 (9)	−0.0063 (10)
C64	0.0259 (11)	0.0259 (10)	0.0538 (14)	−0.0008 (8)	0.0049 (9)	0.0004 (9)
C65	0.0276 (11)	0.0337 (11)	0.0198 (10)	−0.0015 (8)	−0.0007 (8)	−0.0072 (8)
C66	0.0340 (12)	0.0375 (12)	0.0263 (11)	−0.0018 (9)	0.0048 (9)	0.0044 (9)

### Geometric parameters (Å, °)

**Table:**

S1—O2	1.4257 (13)	S2—O6	1.4292 (14)
S1—O3	1.4315 (13)	S2—O5	1.4304 (14)
S1—O1	1.5674 (13)	S2—O4	1.5671 (13)
S1—C5	1.7593 (18)	S2—C38	1.7617 (19)
O1—C8	1.479 (2)	O4—C41	1.483 (2)
C1—C2	1.504 (3)	C34—C35	1.507 (3)
C1—H1A	0.9800	C34—H34A	0.9800
C1—H1B	0.9800	C34—H34B	0.9800
C1—H1C	0.9800	C34—H34C	0.9800
C2—C3	1.392 (3)	C35—C40	1.393 (3)
C2—C7	1.398 (3)	C35—C36	1.394 (3)
C3—C4	1.389 (3)	C36—C37	1.390 (3)
C3—H3	0.9500	C36—H36	0.9500
C4—C5	1.386 (2)	C37—C38	1.386 (3)
C4—H4	0.9500	C37—H37	0.9500
C5—C6	1.390 (3)	C38—C39	1.389 (3)
C6—C7	1.382 (3)	C39—C40	1.394 (3)
C6—H6	0.9500	C39—H39	0.9500
C7—H7	0.9500	C40—H40	0.9500
C8—C9	1.510 (3)	C41—C42	1.505 (3)
C8—C13	1.519 (3)	C41—C46	1.518 (3)
C8—H8	1.0000	C41—H41	1.0000
C9—C10	1.530 (3)	C42—C43	1.530 (2)
C9—H9A	0.9900	C42—H42A	0.9900
C9—H9B	0.9900	C42—H42B	0.9900
C10—C11	1.549 (2)	C43—C44	1.541 (2)
C10—H10A	0.9900	C43—H43A	0.9900
C10—H10B	0.9900	C43—H43B	0.9900
C11—C12	1.526 (2)	C44—C45	1.528 (2)
C11—C11A	1.544 (2)	C44—C44A	1.547 (2)
C11—C17	1.556 (2)	C44—C50	1.555 (2)
C11A—H11A	0.9800	C44A—H44A	0.9800
C11A—H11B	0.9800	C44A—H44B	0.9800
C11A—H11C	0.9800	C44A—H44C	0.9800
C12—C14	1.335 (2)	C45—C47	1.331 (2)
C12—C13	1.519 (3)	C45—C46	1.517 (3)
C13—H13A	0.9900	C46—H46A	0.9900
C13—H13B	0.9900	C46—H46B	0.9900
C14—C15	1.502 (2)	C47—C48	1.503 (2)
C14—H14	0.9500	C47—H47	0.9500
C15—C16	1.536 (2)	C48—C49	1.534 (2)
C15—H15A	0.9900	C48—H48A	0.9900
C15—H15B	0.9900	C48—H48B	0.9900
C16—C21	1.523 (2)	C49—C54	1.528 (2)
C16—C17	1.537 (2)	C49—C50	1.539 (2)
C16—H16	1.0000	C49—H49	1.0000
C17—C18	1.538 (2)	C50—C51	1.536 (2)
C17—H17	1.0000	C50—H50	1.0000
C18—C19	1.538 (2)	C51—C52	1.542 (2)
C18—H18A	0.9900	C51—H51A	0.9900
C18—H18B	0.9900	C51—H51B	0.9900
C19—C20	1.532 (2)	C52—C53	1.535 (2)
C19—H19A	0.9900	C52—H52A	0.9900
C19—H19B	0.9900	C52—H52B	0.9900
C20—C20A	1.536 (2)	C53—C53A	1.542 (2)
C20—C21	1.547 (2)	C53—C54	1.545 (2)
C20—C24	1.557 (2)	C53—C57	1.552 (2)
C20A—H20A	0.9800	C53A—H53A	0.9800
C20A—H20B	0.9800	C53A—H53B	0.9800
C20A—H20C	0.9800	C53A—H53C	0.9800
C21—C22	1.527 (2)	C54—C55	1.533 (3)
C21—H21	1.0000	C54—H54	1.0000
C22—C23	1.546 (3)	C55—C56	1.550 (3)
C22—H22A	0.9900	C55—H55A	0.9900
C22—H22B	0.9900	C55—H55B	0.9900
C23—C24	1.556 (3)	C56—C57	1.552 (3)
C23—H23A	0.9900	C56—H56A	0.9900
C23—H23B	0.9900	C56—H56B	0.9900
C24—C25	1.543 (3)	C57—C58	1.542 (2)
C24—H24	1.0000	C57—H57	1.0000
C25—C26	1.508 (3)	C58—C59	1.508 (2)
C25—C25A	1.539 (3)	C58—C58A	1.533 (3)
C25—H25	1.0000	C58—H58	1.0000
C25A—H25A	0.9800	C58A—H58A	0.9800
C25A—H25B	0.9800	C58A—H58B	0.9800
C25A—H25C	0.9800	C58A—H58C	0.9800
C26—C27	1.322 (3)	C59—C60	1.324 (2)
C26—H26	0.9500	C59—H59	0.9500
C27—C28	1.503 (2)	C60—C61	1.506 (2)
C27—H27	0.9500	C60—H60	0.9500
C28—C29	1.542 (3)	C61—C65	1.536 (3)
C28—C32	1.544 (3)	C61—C62	1.541 (3)
C28—H28	1.0000	C61—H61	1.0000
C29—C30	1.531 (3)	C62—C64	1.522 (3)
C29—C31	1.534 (3)	C62—C63	1.523 (3)
C29—H29	1.0000	C62—H62	1.0000
C30—H30A	0.9800	C63—H63A	0.9800
C30—H30B	0.9800	C63—H63B	0.9800
C30—H30C	0.9800	C63—H63C	0.9800
C31—H31A	0.9800	C64—H64A	0.9800
C31—H31B	0.9800	C64—H64B	0.9800
C31—H31C	0.9800	C64—H64C	0.9800
C32—C33	1.518 (3)	C65—C66	1.518 (3)
C32—H32A	0.9900	C65—H65A	0.9900
C32—H32B	0.9900	C65—H65B	0.9900
C33—H33A	0.9800	C66—H66A	0.9800
C33—H33B	0.9800	C66—H66B	0.9800
C33—H33C	0.9800	C66—H66C	0.9800
			
O2—S1—O3	119.33 (8)	O6—S2—O5	119.26 (8)
O2—S1—O1	104.43 (7)	O6—S2—O4	104.35 (7)
O3—S1—O1	110.39 (8)	O5—S2—O4	109.84 (8)
O2—S1—C5	109.95 (8)	O6—S2—C38	109.82 (9)
O3—S1—C5	108.60 (8)	O5—S2—C38	108.38 (8)
O1—S1—C5	102.86 (8)	O4—S2—C38	104.11 (8)
C8—O1—S1	117.46 (11)	C41—O4—S2	118.40 (11)
C2—C1—H1A	109.5	C35—C34—H34A	109.5
C2—C1—H1B	109.5	C35—C34—H34B	109.5
H1A—C1—H1B	109.5	H34A—C34—H34B	109.5
C2—C1—H1C	109.5	C35—C34—H34C	109.5
H1A—C1—H1C	109.5	H34A—C34—H34C	109.5
H1B—C1—H1C	109.5	H34B—C34—H34C	109.5
C3—C2—C7	118.71 (17)	C40—C35—C36	118.45 (18)
C3—C2—C1	120.74 (18)	C40—C35—C34	120.45 (18)
C7—C2—C1	120.55 (18)	C36—C35—C34	121.08 (18)
C4—C3—C2	120.85 (17)	C37—C36—C35	121.01 (17)
C4—C3—H3	119.6	C37—C36—H36	119.5
C2—C3—H3	119.6	C35—C36—H36	119.5
C5—C4—C3	119.17 (17)	C38—C37—C36	119.37 (17)
C5—C4—H4	120.4	C38—C37—H37	120.3
C3—C4—H4	120.4	C36—C37—H37	120.3
C4—C5—C6	121.14 (17)	C37—C38—C39	121.01 (17)
C4—C5—S1	120.04 (14)	C37—C38—S2	119.81 (14)
C6—C5—S1	118.82 (14)	C39—C38—S2	119.16 (14)
C7—C6—C5	118.96 (18)	C38—C39—C40	118.78 (17)
C7—C6—H6	120.5	C38—C39—H39	120.6
C5—C6—H6	120.5	C40—C39—H39	120.6
C6—C7—C2	121.17 (18)	C35—C40—C39	121.38 (18)
C6—C7—H7	119.4	C35—C40—H40	119.3
C2—C7—H7	119.4	C39—C40—H40	119.3
O1—C8—C9	107.66 (14)	O4—C41—C42	108.23 (14)
O1—C8—C13	107.31 (15)	O4—C41—C46	107.85 (14)
C9—C8—C13	112.22 (15)	C42—C41—C46	111.52 (15)
O1—C8—H8	109.9	O4—C41—H41	109.7
C9—C8—H8	109.9	C42—C41—H41	109.7
C13—C8—H8	109.9	C46—C41—H41	109.7
C8—C9—C10	110.04 (15)	C41—C42—C43	109.48 (15)
C8—C9—H9A	109.7	C41—C42—H42A	109.8
C10—C9—H9A	109.7	C43—C42—H42A	109.8
C8—C9—H9B	109.7	C41—C42—H42B	109.8
C10—C9—H9B	109.7	C43—C42—H42B	109.8
H9A—C9—H9B	108.2	H42A—C42—H42B	108.2
C9—C10—C11	114.88 (16)	C42—C43—C44	114.46 (15)
C9—C10—H10A	108.5	C42—C43—H43A	108.6
C11—C10—H10A	108.5	C44—C43—H43A	108.6
C9—C10—H10B	108.5	C42—C43—H43B	108.6
C11—C10—H10B	108.5	C44—C43—H43B	108.6
H10A—C10—H10B	107.5	H43A—C43—H43B	107.6
C12—C11—C11A	107.17 (15)	C45—C44—C43	109.69 (14)
C12—C11—C10	110.18 (14)	C45—C44—C44A	107.78 (14)
C11A—C11—C10	109.72 (15)	C43—C44—C44A	109.68 (15)
C12—C11—C17	108.98 (14)	C45—C44—C50	108.97 (14)
C11A—C11—C17	112.24 (14)	C43—C44—C50	108.65 (14)
C10—C11—C17	108.54 (14)	C44A—C44—C50	112.04 (14)
C11—C11A—H11A	109.5	C44—C44A—H44A	109.5
C11—C11A—H11B	109.5	C44—C44A—H44B	109.5
H11A—C11A—H11B	109.5	H44A—C44A—H44B	109.5
C11—C11A—H11C	109.5	C44—C44A—H44C	109.5
H11A—C11A—H11C	109.5	H44A—C44A—H44C	109.5
H11B—C11A—H11C	109.5	H44B—C44A—H44C	109.5
C14—C12—C13	120.64 (16)	C47—C45—C46	120.50 (16)
C14—C12—C11	122.48 (16)	C47—C45—C44	122.27 (16)
C13—C12—C11	116.71 (15)	C46—C45—C44	117.15 (15)
C8—C13—C12	110.98 (15)	C45—C46—C41	111.39 (15)
C8—C13—H13A	109.4	C45—C46—H46A	109.3
C12—C13—H13A	109.4	C41—C46—H46A	109.3
C8—C13—H13B	109.4	C45—C46—H46B	109.3
C12—C13—H13B	109.4	C41—C46—H46B	109.3
H13A—C13—H13B	108.0	H46A—C46—H46B	108.0
C12—C14—C15	124.96 (16)	C45—C47—C48	125.15 (16)
C12—C14—H14	117.5	C45—C47—H47	117.4
C15—C14—H14	117.5	C48—C47—H47	117.4
C14—C15—C16	113.52 (14)	C47—C48—C49	113.49 (14)
C14—C15—H15A	108.9	C47—C48—H48A	108.9
C16—C15—H15A	108.9	C49—C48—H48A	108.9
C14—C15—H15B	108.9	C47—C48—H48B	108.9
C16—C15—H15B	108.9	C49—C48—H48B	108.9
H15A—C15—H15B	107.7	H48A—C48—H48B	107.7
C21—C16—C15	111.21 (14)	C54—C49—C48	111.06 (14)
C21—C16—C17	110.09 (14)	C54—C49—C50	110.31 (14)
C15—C16—C17	109.65 (14)	C48—C49—C50	109.12 (14)
C21—C16—H16	108.6	C54—C49—H49	108.8
C15—C16—H16	108.6	C48—C49—H49	108.8
C17—C16—H16	108.6	C50—C49—H49	108.8
C16—C17—C18	113.36 (14)	C51—C50—C49	112.82 (14)
C16—C17—C11	111.53 (14)	C51—C50—C44	112.92 (14)
C18—C17—C11	113.09 (14)	C49—C50—C44	111.62 (14)
C16—C17—H17	106.1	C51—C50—H50	106.3
C18—C17—H17	106.1	C49—C50—H50	106.3
C11—C17—H17	106.1	C44—C50—H50	106.3
C17—C18—C19	114.33 (15)	C50—C51—C52	114.02 (14)
C17—C18—H18A	108.7	C50—C51—H51A	108.7
C19—C18—H18A	108.7	C52—C51—H51A	108.7
C17—C18—H18B	108.7	C50—C51—H51B	108.7
C19—C18—H18B	108.7	C52—C51—H51B	108.7
H18A—C18—H18B	107.6	H51A—C51—H51B	107.6
C20—C19—C18	111.48 (15)	C53—C52—C51	110.86 (14)
C20—C19—H19A	109.3	C53—C52—H52A	109.5
C18—C19—H19A	109.3	C51—C52—H52A	109.5
C20—C19—H19B	109.3	C53—C52—H52B	109.5
C18—C19—H19B	109.3	C51—C52—H52B	109.5
H19A—C19—H19B	108.0	H52A—C52—H52B	108.1
C19—C20—C20A	110.43 (15)	C52—C53—C53A	110.86 (14)
C19—C20—C21	106.59 (14)	C52—C53—C54	106.80 (14)
C20A—C20—C21	112.48 (15)	C53A—C53—C54	112.19 (14)
C19—C20—C24	116.53 (14)	C52—C53—C57	116.91 (14)
C20A—C20—C24	110.01 (14)	C53A—C53—C57	109.47 (14)
C21—C20—C24	100.44 (13)	C54—C53—C57	100.16 (13)
C20—C20A—H20A	109.5	C53—C53A—H53A	109.5
C20—C20A—H20B	109.5	C53—C53A—H53B	109.5
H20A—C20A—H20B	109.5	H53A—C53A—H53B	109.5
C20—C20A—H20C	109.5	C53—C53A—H53C	109.5
H20A—C20A—H20C	109.5	H53A—C53A—H53C	109.5
H20B—C20A—H20C	109.5	H53B—C53A—H53C	109.5
C16—C21—C22	118.58 (15)	C49—C54—C55	118.65 (15)
C16—C21—C20	114.46 (14)	C49—C54—C53	114.87 (14)
C22—C21—C20	104.56 (14)	C55—C54—C53	104.15 (14)
C16—C21—H21	106.1	C49—C54—H54	106.1
C22—C21—H21	106.1	C55—C54—H54	106.1
C20—C21—H21	106.1	C53—C54—H54	106.1
C21—C22—C23	104.07 (14)	C54—C55—C56	104.04 (15)
C21—C22—H22A	110.9	C54—C55—H55A	110.9
C23—C22—H22A	110.9	C56—C55—H55A	110.9
C21—C22—H22B	110.9	C54—C55—H55B	110.9
C23—C22—H22B	110.9	C56—C55—H55B	110.9
H22A—C22—H22B	109.0	H55A—C55—H55B	109.0
C22—C23—C24	107.21 (14)	C55—C56—C57	106.77 (14)
C22—C23—H23A	110.3	C55—C56—H56A	110.4
C24—C23—H23A	110.3	C57—C56—H56A	110.4
C22—C23—H23B	110.3	C55—C56—H56B	110.4
C24—C23—H23B	110.3	C57—C56—H56B	110.4
H23A—C23—H23B	108.5	H56A—C56—H56B	108.6
C25—C24—C23	111.24 (14)	C58—C57—C56	112.65 (15)
C25—C24—C20	118.58 (15)	C58—C57—C53	118.51 (14)
C23—C24—C20	103.41 (14)	C56—C57—C53	103.44 (14)
C25—C24—H24	107.7	C58—C57—H57	107.2
C23—C24—H24	107.7	C56—C57—H57	107.2
C20—C24—H24	107.7	C53—C57—H57	107.2
C26—C25—C25A	109.16 (16)	C59—C58—C58A	108.23 (15)
C26—C25—C24	110.43 (15)	C59—C58—C57	111.30 (14)
C25A—C25—C24	113.53 (15)	C58A—C58—C57	112.29 (14)
C26—C25—H25	107.8	C59—C58—H58	108.3
C25A—C25—H25	107.8	C58A—C58—H58	108.3
C24—C25—H25	107.8	C57—C58—H58	108.3
C25—C25A—H25A	109.5	C58—C58A—H58A	109.5
C25—C25A—H25B	109.5	C58—C58A—H58B	109.5
H25A—C25A—H25B	109.5	H58A—C58A—H58B	109.5
C25—C25A—H25C	109.5	C58—C58A—H58C	109.5
H25A—C25A—H25C	109.5	H58A—C58A—H58C	109.5
H25B—C25A—H25C	109.5	H58B—C58A—H58C	109.5
C27—C26—C25	124.38 (17)	C60—C59—C58	125.96 (16)
C27—C26—H26	117.8	C60—C59—H59	117.0
C25—C26—H26	117.8	C58—C59—H59	117.0
C26—C27—C28	126.95 (17)	C59—C60—C61	125.34 (16)
C26—C27—H27	116.5	C59—C60—H60	117.3
C28—C27—H27	116.5	C61—C60—H60	117.3
C27—C28—C29	111.71 (15)	C60—C61—C65	109.78 (15)
C27—C28—C32	108.93 (15)	C60—C61—C62	113.62 (16)
C29—C28—C32	111.10 (15)	C65—C61—C62	111.72 (15)
C27—C28—H28	108.3	C60—C61—H61	107.1
C29—C28—H28	108.3	C65—C61—H61	107.1
C32—C28—H28	108.3	C62—C61—H61	107.1
C30—C29—C31	109.07 (16)	C64—C62—C63	110.29 (18)
C30—C29—C28	112.32 (15)	C64—C62—C61	113.65 (16)
C31—C29—C28	112.07 (16)	C63—C62—C61	111.00 (16)
C30—C29—H29	107.7	C64—C62—H62	107.2
C31—C29—H29	107.7	C63—C62—H62	107.2
C28—C29—H29	107.7	C61—C62—H62	107.2
C29—C30—H30A	109.5	C62—C63—H63A	109.5
C29—C30—H30B	109.5	C62—C63—H63B	109.5
H30A—C30—H30B	109.5	H63A—C63—H63B	109.5
C29—C30—H30C	109.5	C62—C63—H63C	109.5
H30A—C30—H30C	109.5	H63A—C63—H63C	109.5
H30B—C30—H30C	109.5	H63B—C63—H63C	109.5
C29—C31—H31A	109.5	C62—C64—H64A	109.5
C29—C31—H31B	109.5	C62—C64—H64B	109.5
H31A—C31—H31B	109.5	H64A—C64—H64B	109.5
C29—C31—H31C	109.5	C62—C64—H64C	109.5
H31A—C31—H31C	109.5	H64A—C64—H64C	109.5
H31B—C31—H31C	109.5	H64B—C64—H64C	109.5
C33—C32—C28	114.20 (17)	C66—C65—C61	113.44 (16)
C33—C32—H32A	108.7	C66—C65—H65A	108.9
C28—C32—H32A	108.7	C61—C65—H65A	108.9
C33—C32—H32B	108.7	C66—C65—H65B	108.9
C28—C32—H32B	108.7	C61—C65—H65B	108.9
H32A—C32—H32B	107.6	H65A—C65—H65B	107.7
C32—C33—H33A	109.5	C65—C66—H66A	109.5
C32—C33—H33B	109.5	C65—C66—H66B	109.5
H33A—C33—H33B	109.5	H66A—C66—H66B	109.5
C32—C33—H33C	109.5	C65—C66—H66C	109.5
H33A—C33—H33C	109.5	H66A—C66—H66C	109.5
H33B—C33—H33C	109.5	H66B—C66—H66C	109.5
			
O2—S1—O1—C8	−177.24 (12)	O6—S2—O4—C41	−178.70 (12)
O3—S1—O1—C8	−47.79 (14)	O5—S2—O4—C41	52.34 (14)
C5—S1—O1—C8	67.92 (13)	C38—S2—O4—C41	−63.53 (14)
C7—C2—C3—C4	0.4 (3)	C40—C35—C36—C37	−0.8 (3)
C1—C2—C3—C4	−178.74 (18)	C34—C35—C36—C37	177.58 (17)
C2—C3—C4—C5	−0.1 (3)	C35—C36—C37—C38	0.2 (3)
C3—C4—C5—C6	−0.4 (3)	C36—C37—C38—C39	0.6 (3)
C3—C4—C5—S1	179.51 (14)	C36—C37—C38—S2	179.16 (14)
O2—S1—C5—C4	129.06 (15)	O6—S2—C38—C37	−132.77 (15)
O3—S1—C5—C4	−3.17 (17)	O5—S2—C38—C37	−0.91 (17)
O1—S1—C5—C4	−120.16 (15)	O4—S2—C38—C37	115.98 (15)
O2—S1—C5—C6	−51.05 (17)	O6—S2—C38—C39	45.84 (16)
O3—S1—C5—C6	176.72 (14)	O5—S2—C38—C39	177.70 (14)
O1—S1—C5—C6	59.72 (16)	O4—S2—C38—C39	−65.41 (16)
C4—C5—C6—C7	0.4 (3)	C37—C38—C39—C40	−0.7 (3)
S1—C5—C6—C7	−179.44 (15)	S2—C38—C39—C40	−179.27 (14)
C5—C6—C7—C2	−0.1 (3)	C36—C35—C40—C39	0.7 (3)
C3—C2—C7—C6	−0.4 (3)	C34—C35—C40—C39	−177.70 (17)
C1—C2—C7—C6	178.82 (19)	C38—C39—C40—C35	0.1 (3)
S1—O1—C8—C9	113.97 (14)	S2—O4—C41—C42	116.19 (14)
S1—O1—C8—C13	−125.04 (13)	S2—O4—C41—C46	−123.02 (13)
O1—C8—C9—C10	175.44 (15)	O4—C41—C42—C43	177.69 (14)
C13—C8—C9—C10	57.6 (2)	C46—C41—C42—C43	59.2 (2)
C8—C9—C10—C11	−54.0 (2)	C41—C42—C43—C44	−57.0 (2)
C9—C10—C11—C12	46.7 (2)	C42—C43—C44—C45	48.1 (2)
C9—C10—C11—C11A	−71.1 (2)	C42—C43—C44—C44A	−70.12 (19)
C9—C10—C11—C17	165.97 (15)	C42—C43—C44—C50	167.10 (14)
C11A—C11—C12—C14	−100.7 (2)	C43—C44—C45—C47	139.53 (17)
C10—C11—C12—C14	140.00 (18)	C44A—C44—C45—C47	−101.10 (19)
C17—C11—C12—C14	21.0 (2)	C50—C44—C45—C47	20.7 (2)
C11A—C11—C12—C13	74.55 (19)	C43—C44—C45—C46	−43.9 (2)
C10—C11—C12—C13	−44.8 (2)	C44A—C44—C45—C46	75.46 (19)
C17—C11—C12—C13	−163.77 (15)	C50—C44—C45—C46	−162.74 (14)
O1—C8—C13—C12	−173.35 (14)	C47—C45—C46—C41	−135.25 (18)
C9—C8—C13—C12	−55.3 (2)	C44—C45—C46—C41	48.1 (2)
C14—C12—C13—C8	−134.95 (18)	O4—C41—C46—C45	−173.52 (14)
C11—C12—C13—C8	49.7 (2)	C42—C41—C46—C45	−54.8 (2)
C13—C12—C14—C15	−174.45 (16)	C46—C45—C47—C48	−176.09 (17)
C11—C12—C14—C15	0.6 (3)	C44—C45—C47—C48	0.4 (3)
C12—C14—C15—C16	8.2 (3)	C45—C47—C48—C49	9.2 (3)
C14—C15—C16—C21	−160.27 (14)	C47—C48—C49—C54	−161.03 (15)
C14—C15—C16—C17	−38.3 (2)	C47—C48—C49—C50	−39.2 (2)
C21—C16—C17—C18	−46.6 (2)	C54—C49—C50—C51	−47.0 (2)
C15—C16—C17—C18	−169.27 (14)	C48—C49—C50—C51	−169.30 (15)
C21—C16—C17—C11	−175.61 (13)	C54—C49—C50—C44	−175.44 (14)
C15—C16—C17—C11	61.72 (18)	C48—C49—C50—C44	62.29 (18)
C12—C11—C17—C16	−51.84 (18)	C45—C44—C50—C51	179.78 (14)
C11A—C11—C17—C16	66.72 (18)	C43—C44—C50—C51	60.30 (18)
C10—C11—C17—C16	−171.84 (14)	C44A—C44—C50—C51	−61.04 (19)
C12—C11—C17—C18	179.01 (15)	C45—C44—C50—C49	−51.86 (18)
C11A—C11—C17—C18	−62.44 (19)	C43—C44—C50—C49	−171.34 (14)
C10—C11—C17—C18	59.00 (18)	C44A—C44—C50—C49	67.32 (18)
C16—C17—C18—C19	46.6 (2)	C49—C50—C51—C52	48.9 (2)
C11—C17—C18—C19	174.85 (15)	C44—C50—C51—C52	176.59 (14)
C17—C18—C19—C20	−52.9 (2)	C50—C51—C52—C53	−55.0 (2)
C18—C19—C20—C20A	−65.06 (19)	C51—C52—C53—C53A	−64.76 (18)
C18—C19—C20—C21	57.39 (18)	C51—C52—C53—C54	57.74 (18)
C18—C19—C20—C24	168.49 (15)	C51—C52—C53—C57	168.83 (14)
C15—C16—C21—C22	−57.8 (2)	C48—C49—C54—C55	−59.8 (2)
C17—C16—C21—C22	−179.55 (15)	C50—C49—C54—C55	179.12 (15)
C15—C16—C21—C20	177.98 (14)	C48—C49—C54—C53	176.18 (15)
C17—C16—C21—C20	56.24 (18)	C50—C49—C54—C53	55.06 (19)
C19—C20—C21—C16	−61.51 (18)	C52—C53—C54—C49	−60.29 (18)
C20A—C20—C21—C16	59.65 (19)	C53A—C53—C54—C49	61.37 (19)
C24—C20—C21—C16	176.56 (14)	C57—C53—C54—C49	177.40 (14)
C19—C20—C21—C22	167.10 (14)	C52—C53—C54—C55	168.28 (14)
C20A—C20—C21—C22	−71.74 (18)	C53A—C53—C54—C55	−70.06 (18)
C24—C20—C21—C22	45.18 (16)	C57—C53—C54—C55	45.97 (17)
C16—C21—C22—C23	−161.85 (15)	C49—C54—C55—C56	−161.69 (15)
C20—C21—C22—C23	−32.90 (18)	C53—C54—C55—C56	−32.50 (18)
C21—C22—C23—C24	7.61 (19)	C54—C55—C56—C57	6.2 (2)
C22—C23—C24—C25	148.40 (15)	C55—C56—C57—C58	151.20 (15)
C22—C23—C24—C20	20.06 (18)	C55—C56—C57—C53	22.03 (18)
C19—C20—C24—C25	82.57 (19)	C52—C53—C57—C58	78.6 (2)
C20A—C20—C24—C25	−44.1 (2)	C53A—C53—C57—C58	−48.5 (2)
C21—C20—C24—C25	−162.83 (15)	C54—C53—C57—C58	−166.58 (15)
C19—C20—C24—C23	−153.79 (15)	C52—C53—C57—C56	−155.95 (15)
C20A—C20—C24—C23	79.55 (17)	C53A—C53—C57—C56	76.96 (17)
C21—C20—C24—C23	−39.18 (16)	C54—C53—C57—C56	−41.10 (16)
C23—C24—C25—C26	53.5 (2)	C56—C57—C58—C59	56.8 (2)
C20—C24—C25—C26	173.16 (15)	C53—C57—C58—C59	177.69 (15)
C23—C24—C25—C25A	176.46 (17)	C56—C57—C58—C58A	178.32 (15)
C20—C24—C25—C25A	−63.9 (2)	C53—C57—C58—C58A	−60.8 (2)
C25A—C25—C26—C27	117.9 (2)	C58A—C58—C59—C60	112.9 (2)
C24—C25—C26—C27	−116.6 (2)	C57—C58—C59—C60	−123.2 (2)
C25—C26—C27—C28	−177.30 (17)	C58—C59—C60—C61	−173.26 (17)
C26—C27—C28—C29	−123.9 (2)	C59—C60—C61—C65	115.6 (2)
C26—C27—C28—C32	113.0 (2)	C59—C60—C61—C62	−118.5 (2)
C27—C28—C29—C30	45.2 (2)	C60—C61—C62—C64	−66.8 (2)
C32—C28—C29—C30	167.11 (16)	C65—C61—C62—C64	58.0 (2)
C27—C28—C29—C31	168.45 (16)	C60—C61—C62—C63	58.2 (2)
C32—C28—C29—C31	−69.7 (2)	C65—C61—C62—C63	−176.96 (17)
C27—C28—C32—C33	−63.3 (2)	C60—C61—C65—C66	−71.4 (2)
C29—C28—C32—C33	173.28 (18)	C62—C61—C65—C66	161.63 (17)

### Hydrogen-bond geometry (Å, °)

**Table:**

*D*—H···*A*	*D*—H	H···*A*	*D*···*A*	*D*—H···*A*
C7—H7···O3^i^	0.95	2.49	3.217 (2)	134
C13—H13b···O6	0.99	2.54	3.519 (2)	172
C40—H40···O5^ii^	0.95	2.48	3.193 (2)	131
C42—H42a···O2	0.99	2.56	3.548 (2)	175
C44a—H44c···O2	0.98	2.54	3.390 (2)	145

Symmetry codes: (i) *x*−1, *y*, *z*;  (ii) *x*+1, *y*, *z*.
